# From Molecular Interactions to Solubility in Deep Eutectic Solvents: Exploring Flufenamic Acid in Choline-Chloride- and Menthol-Based Systems

**DOI:** 10.3390/molecules30163434

**Published:** 2025-08-20

**Authors:** Piotr Cysewski, Tomasz Jeliński, Oliwia Kukwa, Maciej Przybyłek

**Affiliations:** Department of Physical Chemistry, Pharmacy Faculty, Collegium Medicum of Bydgoszcz, Nicolaus Copernicus University in Toruń, Kurpińskiego 5, 85-096 Bydgoszcz, Poland; tomasz.jelinski@cm.umk.pl (T.J.); 310389@stud.umk.pl (O.K.); m.przybylek@cm.umk.pl (M.P.)

**Keywords:** flufenamic acid, solubility, deep eutectic solvents, choline chloride, menthol, molecular interactions

## Abstract

This study explores how intermolecular interactions govern the composition of saturated solutions of influence flufenamic acid (FlA) in deep eutectic solvents (DESs). Using choline chloride (ChCl) or menthol (Men) as the HBAs and various polyols as the HBDs, FlA solubility was measured in different DES systems. The experimental values along with intermolecular interactions quantified via COSMOtherm-derived Gibbs free energies were used in the determination of component distributions for varying DES formulations. It was inferred that DES systems primarily consist of molecular complexes (dimers and hetero-pairs) rather than monomers due to their high association propensity. In the case of ChCl-based DESs, the HBA–HBD hetero-pairs are favored and strongly dominate. In contrast, Men-based DESs exhibited a strong attraction to HBDs; however, their self-association led to the predominance of HBD dimers. Solubility of FlA correlated with solute-containing hetero-pairs, peaking at optimal HBA–HBD ratios. These insights support in developing a rationale for DES design for pharmaceutical applications. The conclusions of this study were inferred from a novel crafted physically constrained iterative algorithm that reliably determines molecular composition from the equilibrium constants, overcoming the limitations of conventional numerical solvers in highly associated systems.

## 1. Introduction

Flufenamic acid (FlA) is a non-steroidal anti-inflammatory drug (NSAID) belonging to anthranilic acid derivatives, commonly referred to as fenamates. It features a biphenyl structure bearing a carboxylic acid group and a substituted aniline moiety, i.e., 2-[(3-trifluoromethyl)phenylamino]benzoic acid [1]. This molecular architecture endows FlA with amphiphilic characteristics, where the aromatic rings provide hydrophobicity while the carboxyl and amine groups offer polar interaction sites. Pharmacologically, FlA exhibits anti-inflammatory, analgesic, and antipyretic effects primarily through inhibition of cyclooxygenase (COX) enzymes, which block prostaglandin biosynthesis [2,3]. Additionally, FlA modulates ion channels such as transient receptor potential (TRP) channels and chloride channels, contributing to its expanding profile in neuroscience and cancer research [4,5]. Despite these promising therapeutic potentials, its clinical utility is restricted by poor aqueous solubility, leading to limited bioavailability and erratic absorption profiles [6,7]. According to the Biopharmaceutics Classification System (BCS) [8], FlA is assigned to Class II, characterized by low aqueous solubility and high membrane permeability. The latter property underlies its potential use in transdermal drug delivery systems [9,10].

Deep eutectic solvents (DESs) have emerged as a promising class of green and sustainable solvents, gaining significant attention across various scientific and industrial domains due to their advantageous properties, including tunable physicochemical characteristics, biodegradability, and cost-effectiveness [11,12,13,14,15]. Comprising a mixture of a hydrogen bond acceptor (HBA) and a hydrogen bond donor (HBD), DESs exhibit a melting point that is significantly lower than those of their individual components [16,17]. Natural deep eutectic solvents (NADESs), a subcategory of DESs, are formed from naturally occurring, biodegradable, and often GRAS-certified compounds such as sugars, organic acids, amino acids, and choline derivatives [17,18]. While DESs share several properties with ionic liquids, such as low volatility, high thermal stability, and tunable polarity, they are generally cheaper, less toxic, and easier to prepare; they are often prepared via simple mixing without the need for complex purification [18,19,20]. Their unique ability to form extensive hydrogen bonding networks has positioned them as attractive alternatives to conventional organic solvents, particularly in pharmaceutical applications [21,22,23,24,25,26,27,28]. As has been highlighted, a major challenge for the pharmaceutical industry lies in enhancing the solubility of poorly water-soluble active pharmaceutical ingredients. DESs offer a potential solution to this problem, demonstrating remarkable solubilization capabilities for a wide range of APIs [29,30].

Solubility enhancement in DESs is governed by a combination of specific molecular interactions and broader physicochemical factors. Among the most influential are strong intermolecular interactions, including hydrogen bonding, van der Waals forces, and electrostatic interactions, that occur between APIs’ and DESs’ constituents [31,32,33,34,35,36,37]. The efficient solvation of the solute by molecular assemblies formed within DESs hinders intermolecular aggregation and thereby reduces the risk of precipitation, as confirmed by molecular dynamics simulations [34,38]. However, the solubility of organic compounds in DESs is not yet fully understood and remains highly system-specific. Experimental studies have shown that the enthalpy of dissolution can be either exothermic or endothermic depending on the solute and DES composition [39,40,41,42,43,44,45].

Despite the growing interest and demonstrated utility of DESs in drug delivery and solubilization, a detailed understanding of the specific intermolecular interactions governing the composition of saturated API–DES systems remains crucial. The complex interplay between the HBA, HBD, and the API, especially at varying stoichiometric ratios, can profoundly influence the molecular landscape and, consequently, the API’s solubility at equilibrium. While DES systems can, in principle, be represented by nine distinct mole fraction profiles encompassing monomers, dimers, and hetero-molecular complexes, the strong affinities between constituents often lead to monomer mole fractions being very close to zero, suggesting a predominance of complex formation. FlA is known to exhibit a strong tendency toward dimer formation, driven by π–π stacking and intermolecular hydrogen bonding, particularly in non-polar or weakly interacting solvents [46]. Nevertheless, the nature of its interactions with deep eutectic solvents (DESs) remains largely unexplored, and it is still unclear how the resulting solute–solvent assemblies are organized and how they contribute to API solubilization, especially under saturation conditions. This study aims to comprehensively investigate how intermolecular interactions govern the composition of saturated API–DES systems, focusing on flufenamic acid (FlA), which is a representative poorly soluble API. We have employed a combined experimental and theoretical approach to measure the solubility of FlA in various DES formulations. Specifically, we utilized choline chloride (ChCl) and menthol (Men) as HBAs, paired with glycerol (GLY), tetraethylene glycol (TRG), and other polyols as HBDs. A broad range of HBA–HBD ratios was explored, including 1:1, 1:2, and 1:3 for ChCl-based systems, and 2:1 and 3:1 for menthol-based systems. Computational methods, specifically COSMOtherm, were used to quantify the Gibbs free energies of all possible pair formations, which were then integrated into a Python-based model (Python software, version 3.13) to determine the equilibrium mole fractions of various species. The primary objectives of this manuscript are the following:

1. To quantify and provide detailed concentration profiles of the most abundant species present in saturated DES systems across various HBA–HBD ratios.
2. To investigate the less abundant solute-containing species and establish a direct correlation between their concentrations and the observed FlA solubility.
3. To elucidate the distinct intermolecular interaction patterns and complex formation behaviors in menthol-based versus choline-chloride-based DESs and their direct impact on FlA solubilization.
4. To identify optimal HBA–HBD ratios that maximize the formation of FlA-containing complexes, thereby enhancing API solubility.

By meticulously analyzing the distribution of molecular species, this work not only provides a deeper understanding of the fundamental mechanisms underpinning FlA solubility in DESs but also offers a mechanistic interpretation of experimental solubility data based on molecular-level interactions. The combined use of experimental measurements and COSMO-derived equilibrium modeling establishes a direct link between solute–solvent complexation and solubility outcomes. This integrative approach contributes valuable insights for the rational design of advanced pharmaceutical formulations involving DESs.

## 2. Results and Discussion

This study integrates both experimental and computational approaches to investigate the solubility behavior of FlA in considered DESs. The workflow comprises the following stages:

- Experimental determination of flufenamic acid solubility across a broad range of deep eutectic solvent formulations, differing in hydrogen bond donor/acceptor type and composition, with particular emphasis on their relevance to pharmaceutical applications.
- Thermodynamic characterization of intermolecular interactions using COSMOtherm-derived descriptors, providing molecular-level insight into solute–solvent affinities and dominant association patterns within the systems.
- Application of a physically informed iterative algorithm to infer the equilibrium composition of saturated systems from computed association constants, ensuring chemically consistent distribution of molecular species.
- Interpretation of the predicted molecular distributions in relation to experimental solubility data to elucidate the mechanisms responsible for enhanced solubilization and to identify key interactions that promote the formation of solute-containing complexes.

### 2.1. Saturated FlA–DES Systems

The solubility values expressed in mg/mL are presented in Table S1, (Supplementary Materials). This format enables a direct comparison with previously reported data for water at the same temperature (25 °C), for which the solubility of flufenamic acid is only 9.09 × 10^−3^ mg/mL [47]. Notably, all investigated DESs provided significantly higher solubility compared to water. This finding is particularly relevant for the development of potential oral formulations. In this context, it is important to emphasize that the solubility studies involved compounds that are considered safe and which are already used in oral formulations, including ChCl [48], Men [49,50], and GLY [51,52].

Beyond oral administration, the obtained solubility values may also be of interest for transdermal delivery, where the ability to incorporate the drug into the formulation matrix is equally critical. Considering the drug content per 100 g of solution, the obtained solubility values ranged from 0.03 to 2.46 g (Supplementary Materials, Table S1). While this is below the 3–9% *w*/*w* used in acrylate-based patches [53], it aligns with concentrations that have been shown to enable skin penetration in other topical systems. Wagner et al. (2004) [54] reported effective dermal uptake from formulations containing only 0.45–0.90% *w*/*w* FlA, with the extent of absorption strongly influenced by the choice of vehicle. Similarly, ethosomal gels with 3% *w*/*w* FlA demonstrated superior permeation relative to conventional counterparts, despite moderate drug loading [55].

**Figure 1:**
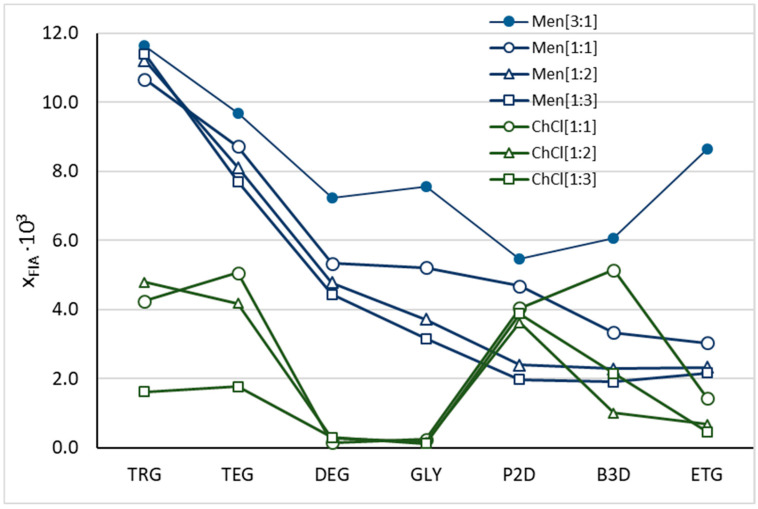
The experimental solubility of flufenamic acid, expressed as the mole fraction, in the studied choline-chloride- (green lines) and menthol-based (blue lines) DESs.

While solubility expressed in mg/mL or % *w*/*w* is useful for formulation comparison and assessing drug loading, mole fraction remains the fundamental unit for thermodynamic modeling and activity-based calculations [56,57,58,59,60,61]. The measured mole fraction solubilities of FLA (X_FlA_) are presented in Figure 1, with detailed values summarized in the Supplementary Materials, Table S2. The highest solubility was observed for the Men/GLY 2:1 system (1.20 × 10^−2^) [47], followed closely by Men/TRG 3:1 (1.16 × 10^−2^). In contrast, DESs based on choline chloride exhibited markedly lower solubilities, with the highest value determined for ChCl/B3D 1:1 (5.14 × 10^−3^).

The lowest solubilities within the ChCl-based series were observed for ChCl/DEG 1:1 (1.55 × 10^−4^) and ChCl/DEG 1:2 (2.86 × 10^−4^); meanwhile, in the menthol-based series, the lowest X_FlA_ values were found for Men/B3D 1:3 (1.90 × 10^−3^) and Men/P2D 1:3 (1.98 × 10^−3^).

### 2.2. Interaction Between DES Components

**Figure 2:**
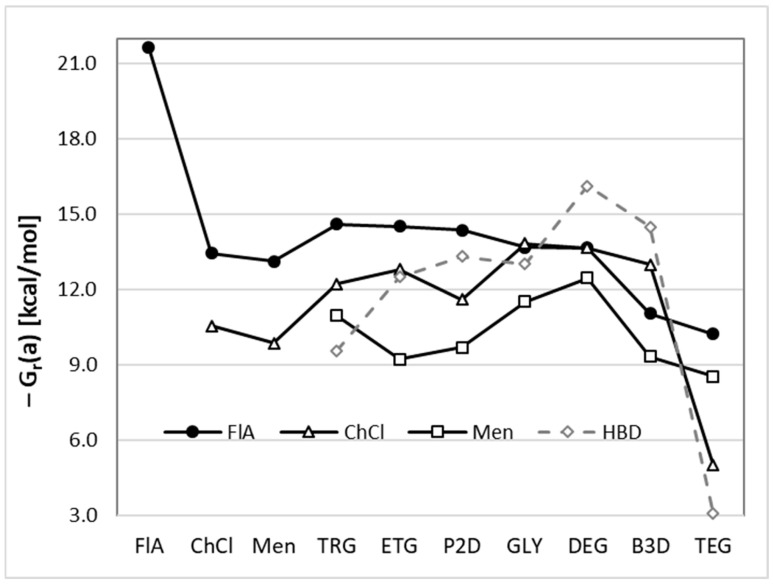
The values of activity-related Gibbs free energy of pair formation reaction between components. The solid line with black circles represents the energetics of all possible FlA pairs formation, solid lines with open triangles and rectangles stand for ΔG_r_(a) of ChCl and Men, respectively. The dashed gray line relates to the self-interactions of HBDs.

The energetics of DES components’ interactions can be characterized by the values of the Gibbs free energy of pair formation ΔG_r_ = –RTln(K_x_·K_γ_), where K_x_ and K_γ_ are mole fractions or activity coefficient products. This property can be attributed both to the self-association of solute or DES components and to the interactions that occur between species. It is worth mentioning that, by definition, ΔG_r_ can be expressed either as a thermodynamic quantity or as a concentration-dependent function. The former is useful for a general way of quantifying interactions as the standard Gibbs free energy change of the reaction (ΔG_r_(a)), and the latter informs us on the actual component propensities at given thermodynamic conditions. Figure 2 presents the ΔG_r_(a) values that quantify the thermodynamics of homo- and hetero-molecular pair formation, involving all possible combinations of solute and solvent molecules in analyzed DESs. Based on the provided plots, the emerging general pattern is quite straightforward; namely, the affinities of FlA are higher than those of ChCl, and these in turn prevail over Men. Additionally, there are some interesting nuances that deserve some deliberation. First, the self-association of flufenamic acid, i.e., its tendency to form dimers, is very high, highlighting the importance of this type of interaction in the studied DESs. All other homo- and hetero-molecular complexes exhibit lower association propensities compared to the FlA dimer. A slightly higher susceptibility for dimer formation was observed for Men compared to ChCl. Taking into account the ΔG_r_ values of hetero-molecular complexes, these two HBA patterns are reversed, meaning that, generally, ChCl exhibits stronger interactions with HBD counterparts compared to Men.

**Figure 3:**
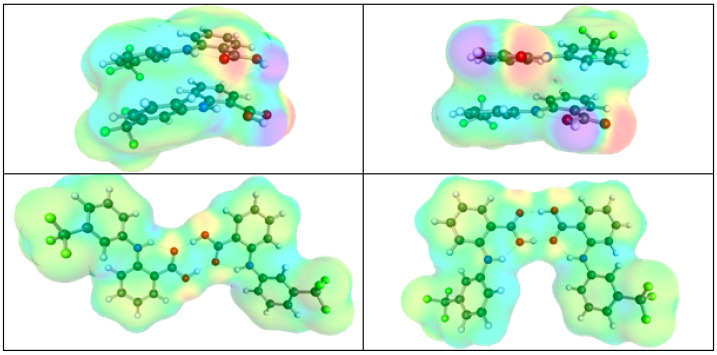
The representation of the most stable conformers of flufenamic acid dimers. Charge density isosurfaces are gradient-colored according to molecular electrostatic potential (MEP): red = negative; blue/violet = positive; green/yellow = neutral.

The observed patterns in the pair interactions of DES constituents do not reveal the structural diversity of the conformers used for intermolecular interaction determinations. It is worth emphasizing that the procedure employed for a representative set of conformers includes not only structures stabilized by hydrogen bonding but also explores the possibility of existing low-energy non-hydrogen bonding contacts. This enables a proper representation of the structural diversity within the studied systems. Indeed, in the case of FlA dimerization, two types of conformers were found, as presented in Figure 3. The most stable ones are stabilized by stacking interactions without forming any hydrogen bonding patterns, and the two FlA molecules are placed in parallel or antiparallel orientations. The second type is a typical motif for many carboxylic acids, which is of the C8(2) type. This decodes the fact that an eight-center ring is stabilized with two hydrogen bonds. The quasi-syn- and anti-conformations can be identified. A diversity of charge density distributions between the two types is noticeable, and stacking does not exclude hydrogen bonding, as these contacts are complementary. Hence, it is very likely that higher-order structures can be formed by both types of bi-molecular patterns of FlA. Such a tendency might be considered the source of the very low solubility of this solute in many solvents due to the formation of larger clusters, which are eventually prone to precipitation.

**Figure 4:**
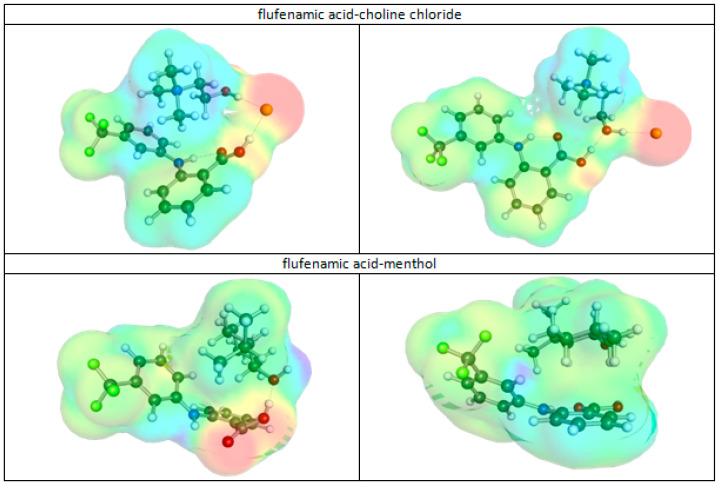
The representation of the most stable conformers of flufenamic acid pairs with HBAs used in this study. Charge density isosurfaces are gradient-colored according to molecular electrostatic potential (MEP): red = negative; blue/violet = positive; green/yellow = neutral.

However, in the case of DESs, there are opposite forces stabilizing the saturation at elevated levels. For example, the complex interplay can occur between FlA and either of HBAs, as illustrated in Figure 4, depicting the most representative low-energy pair conformations, with charge density distributions visualized from COSMOtherm-generated COSMO files. For ChCl-containing systems, there is a concurrency of interactions with the chloride anion between FlA and the choline cation. For some conformations, it is the choline cation that forms stronger interactions with Cl^−^, but in other cases, a bi-center hydrogen bond is formed of both FlA and Ch^+^ with chloride. Apart from this, in many conformations, there are significant contributions coming from the π–electrons of the aromatic ring interacting with the choline moiety. Also, menthol strongly interacts with flufenamic acid in a variety of manners, and the two most stable structures are exemplified in Figure 4. Since Men is less polar compared to ChCl, it is expected that dispersive forces will be higher compared to hydrogen bonding interactions. However, in several conformations, there is a direct interaction of the FlA carboxylic group with the hydroxyl centers of Men, which serve as acceptors. These motifs reflect the molecular flexibility of and variability in the favorable intermolecular interactions that stabilize complexes.

**Figure 5:**
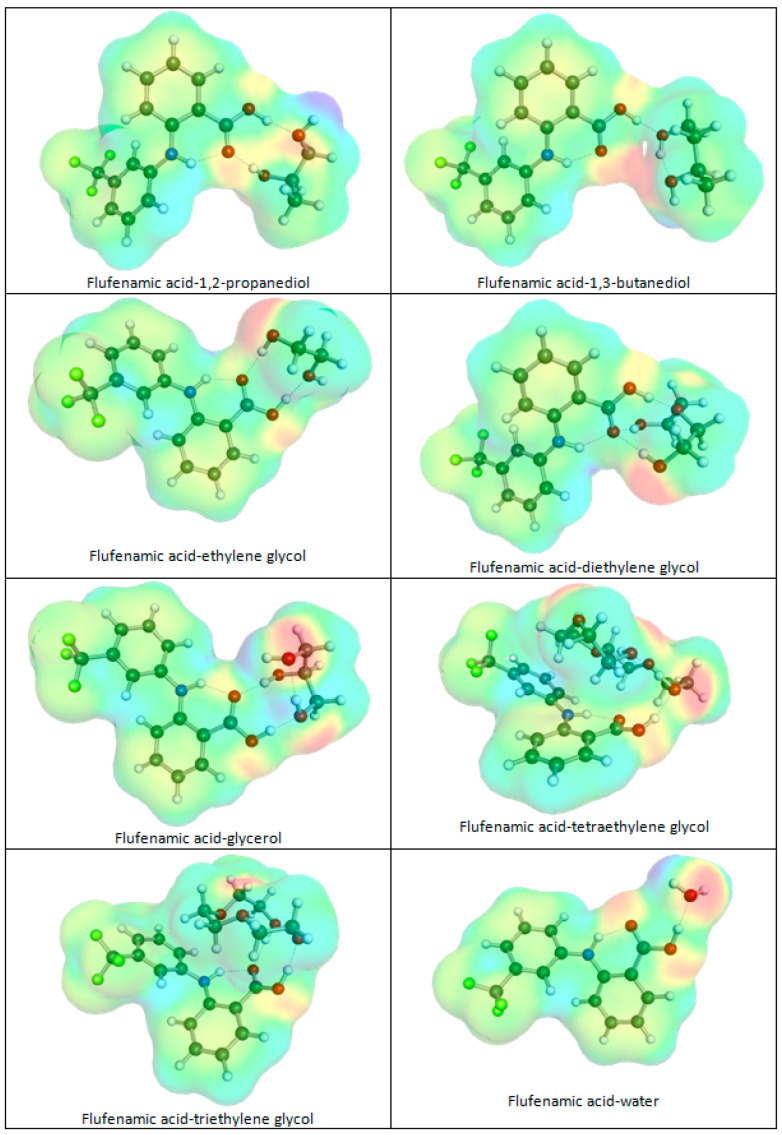
The representation of the most stable conformers of flufenamic acid pairs with HBD used in this study and with water. Charge density isosurfaces are gradient-colored according to molecular electrostatic potential (MEP): red = negative; blue/violet = positive; green/yellow = neutral.

Furthermore, solubility increases in DESs compared to non-DES solvents; this can be attributed also to the strong interactions of flufenamic acid with either of the HBDs utilized in this study, as presented in Figure 5. Typically, hydrogen bonding networks are possible due to the multiple hydroxyl groups that are present on polyols or polyethers. These interactions stabilize both structures of all HBDs due to intramolecular hydrogen bonding as well as intermolecular cluster formation. Smaller polyols, like P2D, TEG, and GLY, are able to form simple patterns of two hydrogen bonds, with the carboxylic group serving as both acceptor and donor at the same time. This is not the case for B3D, for which the internal structure is stabilized by intra-hydrogen bonding, and consequently only one interaction with FlA is allowed. The interactions of the two considered polyethers with flufenamic are much more complex, which might be attributed to the higher flexibility of TEG and TRG. Although both of them can act as acceptors of hydrogen bonding with FlA, additional stabilization comes from non-bonding interactions with both aromatic rings of flufenamic acid. Such interactions offer additional energetic gains compared to polyols, which is probably the reason for the increase in flufenamic acid solubility in DES comprising these kinds of HBDs.

Finally, it is worth mentioning that, in all cases, the intramolecular hydrogen bonds stabilize the flufenamic acid structure formed between the imino-linker and carbonyl group. This makes the structure more rigid, enabling similarity in the hydrogen bonding of FlA across all the DES constituents.

### 2.3. Inferring Molecular Composition from Equilibrium Constants: A Physically Guided Approach

A central challenge in characterizing the molecular composition of API–DES systems lies in translating of equilibrium constants—derived for example from quantum chemical calculations—into physically meaningful mole fractions of species in the solution. While the equilibrium constants for homo-dimer and hetero-pair formation can be computed with high fidelity using COSMOtherm, the inverse problem of reconstructing the system composition from these constants is nontrivial. The difficulty arises from the highly nonlinear nature of equilibrium relationships and the need to simultaneously satisfy mass conservation and stoichiometric restrictions.

Initial attempts to solve this problem employed conventional mathematical solvers, including nonlinear least-squares minimization, root-finding algorithms, and constrained optimization routines such as ‘SLSQP’, ‘east_squares’, or annealing algorithms. Although these methods are mathematically rigorous, they consistently produced physically invalid solutions under realistic conditions—particularly when equilibrium constants spanned several orders of magnitude (e.g., K ≈ 10^−1^ ÷ 10^10^). Common issues included trivial solutions dominated by monomers despite strong association propensities, violation of mass conservation with total mole fractions deviating from unity, and overproduction of associated species that exceeded the available monomer pool.

These failures revealed a critical insight: the problem could not be treated as a purely mathematical inversion. Instead, it required a physically informed algorithm that respects the stoichiometric limits, mass conservation, and thermodynamic constraints inherent to molecular systems. To address this, a hybrid iterative solver was developed that enforces chemical equilibrium through scaled association rather than optimization. The algorithm begins by assigning all material to monomeric species and computes the ideal concentrations of dimers and pairs based on the current monomer pools and the provided equilibrium constants. These ideal values are then scaled globally to ensure that the total consumption of each monomer remains within its available quantity. This scaling step preserves the equilibrium ratios while enforcing mass conservation and preventing overproduction.

The system is updated in a single pass using the scaled equilibrium values, and iterations continue until changes in mole fractions fall below a predefined threshold. Following convergence, all mole fractions are normalized to ensure that the total mole fraction equals unity. The final composition is validated by checking mass balances for each component and verifying that the concentrations of dimers and pairs do not exceed the stoichiometric limits imposed by the available monomers. This approach avoids the rigidity of global solvers and instead mimics the natural relaxation of a chemical system toward equilibrium. It successfully handles extreme equilibrium constants, respects all mass balances, and produces mole fractions that are both numerically stable and chemically plausible.

The final version of the solver was tested across a wide range of DES systems and consistently produced physically valid results. In systems with strong association behavior, such as those involving choline chloride or glycerol, the algorithm correctly predicted the near-complete conversion of monomers into associated species. In more balanced systems, it captured the subtle interplay between homo-dimer and hetero-pair formation. These results confirm that the hybrid iterative solver provides a robust and chemically consistent framework for inferring DES composition from equilibrium constants and offers a practical alternative to conventional numerical methods that often converge to unphysical local minima.

### 2.4. Concentration Profiles in the Saturated Systems

**Figure 6:**
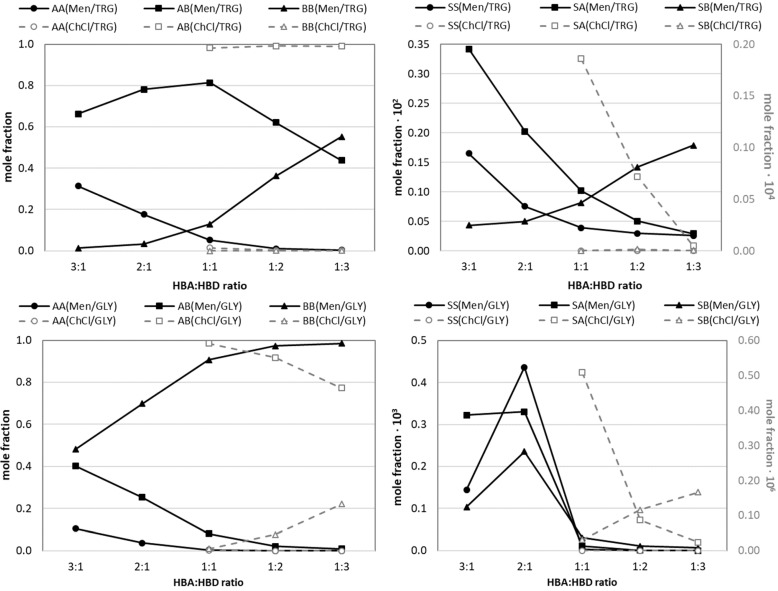
The influence of HBA–HBD ratio on the specific composition of DES systems. The black lines represent menthol-based DESs, and the gray lines correspond to choline-chloride-containing DESs. The plotted values were determined using the Python code using the concentration-dependent values of equilibrium constants of pair formation in TRG- and GLY-based DESs, where S = solute, A = HBA, and B = HBD. The right column presents FlA-related species in analyzed DESs.

In principle, deep eutectic solvent systems can be comprehensively represented by nine distinct mole fraction profiles. These profiles encompass three for monomers (S = solute, A = hydrogen bond acceptor, B = hydrogen bond donor), three for dimers (SS, AA, and BB), and an additional three for hetero-molecular complexes (SA, SB, and AB). Due to the strong association consents between components in all investigated systems, the mole fractions of individual monomers are remarkably close to zero, suggesting that the predominant species in solution are complexes rather than free molecules. Furthermore, the equilibrium concentrations within these systems are not solely dictated by inherent affinities but are significantly influenced by the initial mole fractions of the particular species present. Consequently, HBAs and HBDs are predominantly found in DESs, while the mole fraction of flufenamic acid is a few orders of magnitude lower. Therefore, even though FlA exhibits high association propensity, the concentrations of pairs formed with this solute are considerably lower when compared to pairs formed exclusively by DES constituents. To ensure clarity, the various concentration profiles are presented in separate figures. While all systems underwent detailed investigation, only two exemplary sets of figures are included here. Figure 6 specifically illustrates the systems that demonstrated the highest flufenamic acid solubility, providing valuable insights into the intricate interplay of intermolecular interactions and their impact on the composition of saturated DES systems. Beyond the general trends, a closer look reveals several important patterns and implications. At first, the dominance of complex formation and minimal free monomer content is confirmed. This underscores the idea that these systems are predominantly composed of various complexes rather than free molecules. Figure 6, particularly the left panel depicting the most abundant species, visually confirms this by showing high mole fractions for AA, BB, and AB pairs, with monomeric forms being negligible. In the case of choline-chloride-based DESs, the most striking pattern is the consistent dominance of the hetero-molecular pair (AB), formed between the HBA (ChCl) and HBD (GLY or TRG) across all studied HBA–HBD ratios. This suggests that the primary intermolecular interaction in these systems is the strong association between ChCl and the respective HBD. Additionally, the observed very small decrease in AB concentration with an increase in the amount of either GLY or TRG is noteworthy. Interestingly, in the case of ChCL-GLY, the rise in the HBA–HBD ratio reduces the mole fractions of these hetero-molecular pairs, while GLY-GLY dimerization is systematically rising. The effect is negligible for TRG containing systems.

In the case of menthol-based DESs, ratio-dependent complex formation has much stronger consequences on the component distributions as opposed to ChCl-based DESs. The highest mole fraction for AB pairs is specifically observed at the unimolar (1:1) HBA–HBD proportion for TRG, with a systematic decrease with deviations from this proportion. In the case of GLY, another pattern emerges, as GLY dimers are predominant, irrespective of HBA–HBD ratio, and systematically increase with the rise in HBD amount. Furthermore, the competitive interactions lead to a systematic increase in BB contacts for Men–TRG, which is not the case for GLY-containing systems. Indeed, as the HBD proportion increases relative to menthol, the HBDs begin to self-associate, forming more BB complexes, while menthol’s ability to form self-dimers decreases. It is also interesting to indicate the specific features of TRG. We can observe that the above trends can be attributed to the larger size and potentially lower hydrogen bonding capabilities of TRG. Its longer ether-like chain and increased flexibility might allow for more diverse or stronger self-interactions, but only at higher HBA–HBD ratios. Under such conditions, it might more effectively disrupt menthol’s self-association compared to the smaller GLY molecule.

In the right panel of Figure 6, the mole fraction profiles of the less abundant solute-containing species are plotted (SA, SB, SS). These values are, however, inherently less reliable as they are more prone to numerical noise. Despite this limitation, it is interesting to see the direct link to flufenamic acid solubility. The systems chosen for presentation in Figure 6 were selected based on their highest flufenamic acid solubilization, which can be regarded as indicative of the API’s effective integration into the DES system. In the case of TRG–Men systems, the highest solubility of FlA is associated with high concentrations of hetero-molecular pairs involving the solute and HBA, particularly at the 3:1 TRG–Men ratio. This strongly suggests that the formation of stable complexes between FlA and menthol is the primary mechanism by which FlA is solubilized. The subsequent systematic decrease in all mole fraction values with an increase in the HBD ratio directly reflects the decrease in flufenamic acid solubility in such systems. This highlights that an excess of HBD can be detrimental to FlA solubility in menthol-based systems. On the contrary, low concentrations of FlA in GLY-ChCl systems impose low values of the associated concentrations of FlA species.

This fundamental characteristic has significant implications for how solubility is achieved and sustained in these systems. However, it is important to mention that, due to numerical limitations arising from the very large span of equilibrium constants differing by several orders of magnitude, the quantification of the compositions of FlA-containing pairs is challenging and prone to error. While the algorithm is designed to produce physically consistent mole fractions across a wide range of equilibrium conditions, users should be aware of the inherent limitations arising from the highly nonlinear nature of the system. In particular, the systems with low solute concentrations and weak solute–solvent association are more susceptible to numerical noise and convergence sensitivity. Under such conditions, the equilibrium expressions become shallow and poorly conditioned, making it difficult to distinguish between meaningful association and numerical artifacts. As a result, the computed mole fractions for solute-containing species (especially x_S_, x_SS_, x_SA_, and x_SB_) may exhibit reduced reliability or sensitivity to initial conditions and floating-point precision. These limitations do not affect the overall mass balance or the behavior of dominant solvent–solvent interactions, but they should be considered when interpreting results for systems where solute incorporation is minimal.

## 3. Materials and Methods

### 3.1. Materials

Flufenamic acid (FlA, CAS: 530-78-9, MW = 281.23 g/mol) with a purity of ≥97% was obtained from Sigma Aldrich (Saint Louis, MO, USA). The deep eutectic solvents (DESs) used as solvents in this study comprised a hydrogen bond acceptor (HBA) and a hydrogen bond donor (HBD) in different molar ratios. Two HBAs were used, namely choline chloride (ChCl, CAS: 67-48-1, ≥99%) and menthol (Men, CAS: 89-78-1, ≥98.5%), both purchased from Sigma Aldrich. A total of seven HBDs were included, i.e., ethylene glycol (ETG, CAS: 107-21-1), diethylene glycol (DEG, CAS: 111-46-6), triethylene glycol (TEG, CAS: 112-27-6), tetraethylene glycol (TRG, CAS: 112-60-7), glycerol (GLY, CAS: 56-81-5), 1,2-propanediol (P2D, CAS: 57-55-6), and 1,3-butanediol (B3D, CAS: 107-88-0), similarly delivered by Sigma Aldrich with a purity of ≥99%. Methanol (CAS: 67-56-1, analytical grade) supplied by Chempur (Piekary Śląskie, Poland) was used as a secondary solvent throughout the studies. The above compounds were used as supplied without any additional procedures.

### 3.2. Experimental Solubility Measurements

A well-known and reliable [62,63,64,65] shake-flask method was used for the determination of flufenamic acid solubility in the studied systems. In the first step, deep eutectic solvents were prepared by mixing either ChCl or Men, acting as an HBA, with different HBDs, namely TRG, TEG, DEG, ETG, GLY, P2D, and B3D, in varying molar proportions. The compounds were placed in glass vessels and then heated on a heating plate with simultaneous mixing until a clear homogeneous solution was formed. Next, saturated solutions of FlA were prepared by adding its excess amounts to test tubes containing the DESs prepared earlier. Such samples were placed for 24 h at 25 °C in the Orbital Shaker Incubator ES-20/60 from Biosan (Riga, Latvia) with additional mixing at 60 rpm. The solutions were then filtered with the help of a syringe with a PTFE filter of 0.22 µm pore size. The tubes, syringes, pipette tips, and filters were also kept at 25 °C to avoid precipitation.

A calibration curve for flufenamic acid was obtained by successful dilutions of its initial stock solution in methanol and subsequent spectrophotometric measurements of a set of solutions with decreasing concentrations. The spectra were recorded with an A360 spectrophotometer from AOE Instruments (Shanghai, China) in the wavelength range from 200 nm to 500 nm with a 1 nm resolution. The analytical wavelength was set to 343 nm. The final linear regression equation, obtained after the averaging of the separate calibration curves, was found to be A = 29.148 × C [mg/mL] − 0.019, with the determination coefficient equaling R^2^ = 0.9996. The limit of detection (LOD) and the limit of quantification (LOQ) for the curve were LOD = 1.27 × 10^−3^ mg/mL and LOQ = 3.84 × 10^−3^ mg/mL.

The filtered samples were subjected to spectrophotometric measurements using the same apparatus and the same spectral parameters. Samples were also diluted with methanol accordingly in order to remain in the linearity range of the curve. The spectra of the samples were recorded, and the absorbance values at a characteristic wavelength of 343 nm were used for the determination of flufenamic acid concentration using the prepared calibration curve.

While UV–Vis spectrophotometry is not inherently selective, a control experiment was conducted to assess potential spectral interferences from the DES components. Blank samples were prepared using the same procedure and solvent composition as the test samples, but without the addition of FlA. The measurements confirmed that the DES components did not produce any detectable signal at the selected wavelength.

To calculate the mole fractions of FlA in the saturated solutions, the density of each sample was determined by transferring 1 mL of solution into a 10 mL volumetric flask and weighing it using a RADWAG AS 110.R2 PLUS analytical balance (Radom, Poland) with a precision of 0.1 mg. Three separate measurements were conducted for each system, and the obtained values were averaged.

### 3.3. Intermolecular Interactions Computations

Utilization of the COSMO-RS framework [66] requires, in the first step, the appropriate characteristics of structural diversity of all systems constituents. Since the protocol applied here adheres to previously published schemes [67,68], only a brief synopsis is provided here. The most stable structures of monomeric forms and their associated homo- and hetero-molecular pairs in DES were identified through a comprehensive conformational analysis using COSMOconf and COSMOtherm. For each molecule or complex, up to ten low-energy conformations were determined for both gas and condensed phases, the latter accounting for solvent effects under the conductor-like screening model. The resulting “cosmo” and “energy” files were generated using the BP_TZVPD_FINE_24.ctd parameter set, essential for thermodynamic calculations in COSMOtherm.

The conformational analysis of the deep eutectic solvent components was conducted to generate a diverse set of conformers prior to COSMOtherm calculations. Initial molecular structures of the DES constituents, including either API, HBAs, or HBDs, were subjected to a systematic conformer search using COSMOconf (version 2023, BIOVIA COSMOlogic). This program employs a combination of molecular mechanics, semi-empirical methods, and DFT approaches to explore the conformational space, generating an ensemble of low-energy conformers by systematically varying torsional angles and optimizing geometries. These conformers were then refined using TURBOMOLE (version 7.7, 2023, TURBOMOLE GmbH) at the DFT level with the BP86 functional and def2-TZVP basis set, incorporating the COSMO continuum solvation model to account for solvent effects during geometry optimization and energy calculations. The resulting conformers were ranked by their energies and RMSDs, and a representative subset was selected to ensure adequate coverage of the conformational space while minimizing redundancy. Similarly, to capture the structural diversity of molecular contacts, a detailed conformational analysis was conducted to identify the most stable and probable complexes. The COSMOtherm program facilitated the initial generation of molecular pairs by systematically varying their mutual orientations with a 15° rotation step, using the command “CONTACT = {1 2} ssc_probability ssc_weak ssc_ang = 15.0,” which accounted for both hydrogen bonding and weak interactions. In this step, great variety of structures is generated, which typically are far from optimal. Hence, the full gradient optimizations were performed with TURBOMOLE using the RI-DFT BP86 method. Redundant and high-energy conformers were filtered out based on RMSD and a 2.5 kcal/mol energy threshold relative to the most stable conformer, yielding a refined set of unique contacts for two-molecule complexes. Since COSMOtherm requires conformers optimized in both gas and bulk phases, this process was performed twice. The optimized conformers were converted into “cosmo” and “energy” files, compatible with the BP_TZVPD_FINE_21.ctd parameter set for high-accuracy RI-BP/TZVP//TZVPD-FINE calculations in COSMOtherm.

These optimized structures of monomers and pairs were utilized to compute thermodynamic properties such as the concentration-dependent (ΔG_r_(x) = –RTln(K_x_)) and activity-related (corresponding to the standard state, ΔG_r_(a) = –RTln(K_a_)) values of the Gibbs free energies of bi-molecular complex formation. Both types of values of Gibbs free energy are interrelated due to the fundamental relationship K_a_ = K_x_·K_γ_, where the latter represents the product of activity coefficients. All these characteristics are directly available in the COSMOtherm output. In computations of all thermodynamic properties, the proportions of HBA and HBD were preserved to properly represent the experimentally studied systems. This was achieved by explicitly providing mole fractions in the input files of all components in the monomeric forms. Since there is no available information about the mole fraction of bi-molecular clusters, they were set to zero; this does not affect the standard values. However, this is the source of high diversity in the computed Kx values due to the artificial non-equilibrium state. That is why the Python code was developed for finding optimal compositions, including unknown pairs of mole fractions in the saturated systems.

### 3.4. DES Composition Determination

To determine the concentration profiles, the Python code (Supplementary Materials, “S2. Python Code Solving Mole Fraction Equilibria” subsection) was written for solving a system of nonlinear equations to determine equilibrium mole fractions of monomers (*x_S_*, *x_A_*, *x_B_*) and their associated complexes (*x_SS_*, *x_AA_*, *x_BB_*, *x_SA_*, *x_SB_ x_AB_*) in a ternary chemical system. The system models the self-association and cross-association of components A, B, and S, governed by association constants (*K_AA_*, *K_BB_*, *K_SS_*, *K_AB_*, *K_SA_*, *K_SB_*). The equilibrium mole fractions of complexes are computed as:

$$S=x_{S}+2K_{SS}\cdot x_{S}^{2}+K_{SA}\cdot x_{A}\cdot x_{S}+K_{SB}\cdot x_{B}\cdot x_{S}$$

$$a=x_{A}+2K_{AA}\cdot x_{A}^{2}+K_{AB}\cdot x_{A}\cdot x_{B}+K_{SA}\cdot x_{A}\cdot x_{S}$$

$$b=x_{B}+2K_{BB}\cdot x_{B}^{2}+K_{AB}\cdot x_{A}\cdot x_{B}+K_{SB}\cdot x_{B}\cdot x_{S}$$

where *S*, *a*, and *b* are the total mole fractions in equilibrated systems. In the case of solute, this corresponds to solubility, and for DES components, this corresponds to the total mole fraction of components of solute free state. $x_{A}^{*}$ and $x_{B}^{*}$ were are used for mole fraction determination: *a* = $x_{A}^{*}.(1+S)$ and *b* = $x_{b}^{*}.(1+S)$. The code employs a hybrid iterative solver that enforces chemical equilibrium through physically constrained scaling rather than numerical optimization. The algorithm begins by assigning all material to monomeric species and then computes the ideal concentrations of dimers and pairs based on the current monomer pools and the provided equilibrium constants. These ideal values reflect the thermodynamic drive toward association but are not applied directly. Instead, the total consumption of each monomer is calculated, and a global scaling factor is introduced to ensure that the formation of associated species remains within the bounds of physical availability. This scaling step guarantees that no species is overproduced and that mass conservation is strictly maintained. The system is updated in a single pass using the scaled equilibrium values, and iterations continue until changes in mole fractions fall below a predefined threshold, indicating numerical convergence. Following convergence, all mole fractions are normalized to ensure that the total mole fraction equals unity. This rescaling preserves the equilibrium ratios while enforcing compositional realism. The final composition is validated by checking mass balances for each component and verifying that the concentrations of dimers and pairs do not exceed the stoichiometric limits imposed by the available monomers.

The output includes the mole fractions of all species, along with error metrics quantifying deviations from mass balance and physical feasibility. This approach provides a robust and chemically consistent means of inferring DES composition across a wide range of equilibrium conditions. By avoiding conventional optimization routines, it overcomes the common problem of convergence to local minima that often arises when using standard numerical libraries, especially in systems governed by extreme equilibrium constants. The input data necessary for reproduction were provided in Table S3.

## 4. Conclusions

A comprehensive investigation into the composition of saturated API–DES systems provided important insights into the role of the intermolecular interactions that govern flufenamic acid solubility. Our findings confirm that DES systems are characterized by the overwhelming presence of various molecular complexes rather than free monomers as a consequence of the strong association propensities between their constituents. The distribution of these complexes, including HBA–HBD (AB), HBA–HBA (AA), HBD–HBD (BB), and solute-containing (SA, SB, and SS) pairs, is profoundly influenced by the specific hydrogen bond acceptors (HBAs) and hydrogen bond donors (HBDs) employed, as well as their stoichiometric ratios. A clear distinction emerged between the two classes of DESs studied. Systems based on choline chloride (ChCl) consistently showed a predominance of AB complexes, whose concentrations are loosely related to the proportions of HBA–HBD. In contrast, DESs based on menthol (Men) exhibited more nuanced behavior, with optimal AB complex formation occurring at unimolar HBA–HBD ratios, reflecting a delicate balance of interactions that gives way to increased HBD self-association (BB) at other ratios. Notably, these trends were more pronounced in systems containing tetraethylene glycol (TRG) as the HBD than in those with glycerol (GLY), suggesting its unique structural properties might enhance specific interactions or competitive effects.

Crucially, the solubility of FlA in these DESs is directly correlated with the formation and concentration of specific hetero-molecular pairs involving the solute. The highest FlA solubility observed in the 3:1 GLY-Men system was unequivocally linked to the highest overall concentration of FlA-containing complexes (SA, SB). This highlights that FlA solubilization is primarily achieved through its specific complexation with DES constituents.

This study demonstrated that specific solute-containing complexes (SA, SB) are quantitatively responsible for the solubility patterns observed across various DES formulations. These findings clarify how the relative affinities and self-association tendencies of HBA and HBD components determine the balance between solute–solvent and solvent–solvent complexes. The proposed algorithm, linking COSMOtherm-derived affinities with mole fraction composition, offers a practical tool for predicting optimal HBA–HBD ratios maximizing solute incorporation in DES. Notably, given the widespread use of Men and GLY in topical formulations and the pharmacological relevance of FlA in dermal applications, the investigated DESs represent a promising platform for the development of skin-targeted delivery systems and warrant further investigation.

## Data Availability

All data supporting the reported results are available on request from the corresponding author.

## Supplementary Materials

The following supporting information can be downloaded at: https://www.mdpi.com/article/10.3390/molecules30163434/s1, Table S1. Solubility of flufenamic acid (FlA) expressed in mg/mL in deep eutectic solvents (DESs) composed of choline chloride or menthol as hydrogen bond acceptors (HBAs) and various polyols as hydrogen bond donors (HBDs), determined at 25 °C across different molar ratios. Corresponding FlA contents are shown in parentheses as weight percentages (*w*/*w*), Table S2: Mole fraction solubility of flufenamic acid (FlA) in deep eutectic solvents (DESs) composed of choline chloride or menthol as the hydrogen bond acceptor (HBA) and various polyols as hydrogen bond donors (HBDs) at different molar ratios, measured at 25 °C; “S2. Python Code Solving Mole Fraction Equilibria” section; Table S3: The input data (equilibrium constants) for all studied DES systems.

## Abbreviations

The following abbreviations are used in this manuscript:

AA	hydrogen bond acceptor dimer
AB	hydrogen bond acceptor–hydrogen bond donor pair
API	active pharmaceutical ingredient
B3D	1,3-butanediol
BB	hydrogen bond donor dimer
BCS	Biopharmaceutics Classification System
BP86	Becke–Perdew 1986 functional; GGA-type DFT functional
COSMO-RS	Conductor-like Screening Model for Realistic Solvation
COX	cyclooxygenase
ChCl	choline chloride
DEG	diethylene glycol
DES	deep eutectic solvent
DFT	Density Functional Theory
ETG	ethylene glycol
FlA	flufenamic acid
GLY	glycerol
GRAS	Generally Recognized As Safe
HBA	hydrogen bond acceptor
HBD	hydrogen bond donor
LOD	limit of detection
LOQ	limit of quantification
Men	menthol
NSAID	non-steroidal anti-inflammatory drug
P2D	1,2-propanediol
PTFE	polytetrafluoroethylene
RI-DFT	resolution of the identity density functional theory; approximation accelerating Coulomb integral evaluation in DFT
RMSD	root-mean-square deviation; measure of structural similarity between aligned molecular conformations
SA	solute–hydrogen bond acceptor pair
SB	solute–hydrogen bond donor pair
SLSQP	Sequential Least Squares Programming Method
SS	solute–solute dimer
TEG	triethylene glycol
TRG	tetraethylene glycol
TRP	transient receptor potential
def2-TZVP	split-valence triple-zeta basis set with polarization functions; part of the def2 family developed by Weigend and Ahlrichs
