# Supplementary material for: From Molecular Interactions to Solubility in Deep Eutectic Solvents: Exploring Flufenamic Acid in Choline-Chloride- and Menthol-Based Systems
Source: Molecules. 2025 Aug 20;30(16):3434. doi: 10.3390/molecules30163434 (PMC12388410; doi:10.3390/molecules30163434)
Supplement: Supplementary file 1 [file molecules-30-03434-s001.zip › molecules-3796220-supplementary.pdf]

Supplementary Materials

From Molecular Interactions to Solubility: Exploring Flufenamic Acid in Choline and Menthol DES Systems

Piotr Cysewski, Tomasz Jeliński, Oliwia Kukwa and Maciej Przybyłek

Department of Physical Chemistry, Pharmacy Faculty, Collegium Medicum of Bydgoszcz, Nicolaus Copernicus University in Toruń, Kurpińskiego 5, 85-096 Bydgoszcz, Poland

S1. Experimental data 2

Table S1. Solubility of flufenamic acid (FlA) expressed in mg/mL in deep eutectic solvents (DESs) composed of choline chloride or menthol as hydrogen bond acceptors (HBAs) and various polyols as hydrogen bond donors (HBDs), determined at 25 °C across different molar ratios. Corresponding FlA contents are shown in parentheses as weight percentages (w/w). 2

Table S2. Mole fraction solubility of flufenamic acid (FlA) in deep eutectic solvents (DESs) composed of choline chloride or menthol as the hydrogen bond acceptor (HBA) and various polyols as hydrogen bond donors (HBDs) at different molar ratios, measured at 25 °C. 3

S2. Python Code Solving Mole Fraction Equilibria 4

S2.1. System of Equations 4

S2.2. Input Requirements 4

S2.2. Output 5

S2.3 Python code 5

S2.4. Input data 9

Table S3. The input data (equilibrium constants) for all studied DES systems. 9

S1. Experimental data

Table S1. Solubility of flufenamic acid (FlA) expressed in mg/mL in deep eutectic solvents (DESs) composed of choline chloride or menthol as hydrogen bond acceptors (HBAs) and various polyols as hydrogen bond donors (HBDs), determined at 25 °C across different molar ratios. Corresponding FlA contents are shown in parentheses as weight percentages (w/w).

Solvent	Solubility of FlA				
	**3:1**	**2:1**	**1:1**	**1:2**	**1:3**
**ChCl/P2D**	-	-	10.936±0.517 (1.05±0.05)	11.473±0.545 (1.04±0.05)	13.039±0.669 (1.18±0.06)
**ChCl/DEG**	-	-	0.401±0.014 (0.04±0.00)	0.770±0.037 (0.07±0.00)	0.832±0.046 (0.07±0.00)
**ChCl/TEG**	-	-	11.915±0.534 (0.98±0.04)	9.365±0.464 (0.80±0.04)	3.897±0.159 (0.34±0.01)
**ChCl/B3D**	-	-	14.130±0.558 (1.25±0.05)	2.724±0.121 (0.26±0.01)	6.116±0.227 (0.59±0.02)
**ChCl/GLY**	-	-	0.708±0.034 (0.06±0.00)	0.524±0.021 (0.04±0.00)	0.393±0.017 (0.03±0.00)
**ChCl/ETG**	-	-	4.498±0.148 (0.40±0.01)	2.502±0.092 (0.22±0.01)	1.825±0.091 (0.16±0.01)
**ChCl/TRG**	-	-	8.842±0.461 (0.71±0.04)	8.842±0.461 (0.76±0.04)	2.898±0.113 (0.25±0.01)
**Men/P2D**	10.622±0.438 (1.12±0.05)	10.833±0.566 (1.11±0.06)	11.270±0.594 (1.13±0.06)	6.491±0.273 (0.66±0.03)	5.525±0.312 (0.58±0.03)
**Men/DEG**	13.837±0.572 (1.40±0.06)	19.653±0.915 (1.97±0.09)	11.670±0.485 (1.14±0.05)	11.214±0.358 (1.09±0.03)	10.686±0.471 (1.04±0.05)
**Men/TEG**	17.141±0.734 (1.74±0.07)	18.846±0.686 (1.82±0.07)	16.800±0.643 (1.59±0.06)	15.888±0.721 (1.49±0.07)	15.249±0.820 (1.42±0.08)
**Men/B3D**	11.603±0.563 (1.21±0.06)	13.428±0.562 (1.40±0.06)	7.280±0.336 (0.76±0.03)	5.547±0.240 (0.57±0.02)	4.882±0.188 (0.50±0.02)
**Men/GLY**	14.240±0.669 (1.50±0.07)	23.875±1.025 (2.46±0.11)	11.771±0.397 (1.17±0.04)	9.576±0.491 (0.92±0.05)	9.139±0.347 (0.82±0.03)
**Men/ETG**	17.890±1.000 (1.82±0.10)	12.132±0.733 (1.23±0.07)	7.780±0.319 (0.78±0.03)	7.300±0.278 (0.70±0.03)	7.821±0.403 (0.71±0.04)
**Men/TRG**	20.119±0.772 (1.96±0.08)	18.169±0.898 (1.76±0.09)	17.569±0.908 (1.70±0.09)	17.886±0.741 (1.72±0.07)	18.111±0.970 (1.73±0.09)

Table S2. Mole fraction solubility of flufenamic acid (FlA) in deep eutectic solvents (DESs) composed of choline chloride or menthol as the hydrogen bond acceptor (HBA) and various polyols as hydrogen bond donors (HBDs) at different molar ratios, measured at 25 °C.

Solvent	$x_{FlA}×10^3$				
	**3:1**	**2:1**	**1:1**	**1:2**	**1:3**
**ChCl/P2D**	-	-	4.046±0.199	3.627±0.187	3.881±0.205
**ChCl/DEG**	-	-	0.155±0.005	0.286±0.014	0.302±0.016
**ChCl/TEG**	-	-	5.065±0.250	4.174±0.219	1.772±0.074
**ChCl/B3D**	-	-	5.138±0.228	0.973±0.044	2.155±0.084
**ChCl/GLY**	-	-	0.250±0.011	0.170±0.006	0.121±0.005
**ChCl/ETG**	-	-	1.438±0.052	0.686±0.024	0.462±0.023
**ChCl/TRG**	-	-	4.252±0.211	4.795±0.249	1.625±0.069
**Men/P2D**	5.456±0.215	5.139±0.258	4.683±0.241	2.402±0.104	1.975±0.102
**Men/DEG**	7.224±0.313	9.901±0.495	5.336±0.214	4.782±0.145	4.434±0.214
**Men/TEG**	9.677±0.417	10.050±0.441	8.722±0.334	8.112±0.322	7.698±0.409
**Men/B3D**	6.048±0.294	6.741±0.279	3.329±0.129	2.284±0.087	1.902±0.077
**Men/GLY**	7.553±0.389	11.986±0.534	5.215±0.172	3.716±0.185	3.162±0.125
**Men/ETG**	8.655±0.460	5.520±0.285	3.042±0.139	2.334±0.074	2.162±0.110
**Men/TRG**	11.639±0.498	10.664±0.486	10.665±0.515	11.202±0.503	11.403±0.591

S2. Python Code Solving Mole Fraction Equilibria

The provided Python code computes equilibrium mole fractions of monomers (`x_A`, `x_B`, `x_S`) and their associated species (`x_AA`, `x_BB`, `x_SS`, `x_AB`, `x_SA`, `x_SB`) in a ternary chemical system. The model accounts for both self-association and cross-association among components A, B, and S, governed by six equilibrium constants: K_AA, K_BB, K_SS, K_AB, K_SA, and K_SB. Rather than relying on conventional numerical optimization, the code implements a physically-constrained iterative algorithm that mimics the natural relaxation of the system toward equilibrium. At each step, ideal concentrations of associated species are computed from the current monomer pools and scaled globally to ensure mass conservation and stoichiometric feasibility. The algorithm proceeds until convergence is achieved, and the final mole fractions are normalized to preserve compositional realism. The output includes mole fractions of all species along with error metrics assessing mass balance and physical validity. The code is designed for batch processing and can be applied to a wide range of DES systems with varying component ratios and equilibrium constants.

S2.1. System of Equations

The Python code solves a system of nonlinear equations that describe the equilibrium composition of a ternary chemical system involving components A, B, and S. These components can form homo-dimers (AA, BB, SS) and hetero-pairs (AB, SA, SB), governed by six equilibrium constants: (K_{AA}, K_{BB}, K_{SS}, K_{AB}, K_{SA}, K_{SB}). The system is defined by two sets of relationships:

(i) Equilibrium Conditions: each associated species is formed according to a mass-action equilibrium expression:

$x_{SS} = 2K_{SS} \cdot x_S^2; x_{AA} = 2K_{AA} \cdot x_A^2; x_{BB} = 2K_{BB} \cdot x_B^2;$

$x_{SA} = K_{SA} \cdot x_S \cdot x_A \,; x_{SB} = K_{SB} \cdot x_S \cdot x_B \,;; x_{AB} = K_{AB} \cdot x_A \cdot x_B \,;;$

These expressions define the ideal concentrations of associated species based on the current monomer pools and the equilibrium constants.

(ii) Mass balance constraints and normalization condition

The total amount of each component must be conserved across all species:

$S = x_S + 2K_{SS} \cdot x_S^2 + K_{SA} \cdot x_A \cdot x_S + K_{SB} \cdot x_B \cdot x_S$

$a = x_A + 2K_{AA} \cdot x_A^2 + K_{AB} \cdot x_A \cdot x_B + K_{SA} \cdot x_A \cdot x_S$

$b = x_B + 2K_{BB} \cdot x_B^2 + K_{AB} \cdot x_A \cdot x_B + K_{SB} \cdot x_B \cdot x_S$

These equations ensure that the mole fractions of monomers and their associated forms sum to the initial input values for each component. After convergence, all mole fractions are normalized to ensure the total composition sums to unity:

$$1 = x_S + x_{SS} + x_{SA} + x_{SB} + x_A + x_{AA} + x_{AB} + x_B + x_{BB}$$

This step preserves the equilibrium ratios while enforcing compositional realism.

S2.2. Input Requirements

The code expects a Pandas DataFrame (`data`) with the following columns:

- `a`, `b`, `S`: Initial mole fractions of components A, B, and S (typically (a + b + S = 1)).
- `K_AA`, `K_BB`, `K_SS`: Self-association constants.
- `K_AB`, `K_SA`, `K_SB`: Cross-association constants.

All inputs should be positive, and association constants are assumed to be in consistent units (e.g., L/mol if concentrations are molar).

S2.2. Output

The code generates an Excel file (`results.xlsx`) with the following columns for each data set:

- `Set`: Index of the data set (1-based).
- `a`, `b`, `S`: Input mole fractions.
- `x_A`, `x_B`, `x_S`: Equilibrium monomer mole fractions.
- `x_AA`, `x_BB`, `x_SS`, `x_AB`, `x_SA`, `x_SB`: Equilibrium complex mole fractions.
- `total`: Sum of mole fractions (should be (\approx 1)).
- `obj_value`: Objective function value (sum of squared residuals, ideally (< 10^{-6})).

Warnings for inconsistent solutions, high objective function values, or non-unity mole fraction sums are printed to the console for diagnostic purposes.

S2.3 Python code

```
#!/usr/bin/env python

# coding: utf-8

import numpy as np

import pandas as pd

# data loading

file_path = 'DES_inputK.xlsx'

data = pd.read_excel(file_path, sheet_name='Kx',usecols='A:M')

data.fillna(0, inplace=True)

input_data = data.copy()

input_data = input_data.dropna(how='all')  # Remove empty rows

# main

results = []

MAX_ITER = 1000

TOL = 1e-12

for idx, row in input_data.iterrows():

    S, a, b = row["S"], row["a"], row["b"]

    KSS, KAA, KBB = row["K_SS"], row["K_AA"], row["K_BB"]

    KSA, KSB, KAB = row["K_SA"], row["K_SB"], row["K_AB"]


    # Initial guess: all monomers

    xS = S

    xA = a

    xB = b


    for _ in range(MAX_ITER):
```

```
prev = np.array([xS, xA, xB])

# Compute ideal associated species from current monomers
xSS = KSS * xS**2
xAA = KAA * xA**2
xBB = KBB * xB**2
xSA = KSA * xS * xA
xSB = KSB * xS * xB
xAB = KAB * xA * xB

# Total monomer consumption
used_S = 2*xSS + xSA + xSB
used_A = 2*xAA + xAB + xSA
used_B = 2*xBB + xAB + xSB

# Compute scaling factors to enforce mass conservation
scale_S = S / (xS + used_S)
scale_A = a / (xA + used_A)
scale_B = b / (xB + used_B)
scale = min(scale_S, scale_A, scale_B)

# Apply scaling
xS *= scale
xA *= scale
xB *= scale
xSS *= scale
xAA *= scale
xBB *= scale
xSA *= scale
xSB *= scale
xAB *= scale

# Check convergence
current = np.array([xS, xA, xB])
if np.max(np.abs(current - prev)) < TOL:
    break
```

```
# Normalize
total = xS + xA + xB + xSS + xAA + xBB + xSA + xSB + xAB
xS /= total
xA /= total
xB /= total
xSS /= total
xAA /= total
xBB /= total
xSA /= total
xSB /= total
xAB /= total

S_norm = S / (S + a + b)
a_norm = a / (S + a + b)
b_norm = b / (S + a + b)

err_a = abs(xA + 2*xAA + xAB + xSA - a_norm)
err_b = abs(xB + 2*xBB + xAB + xSB - b_norm)
err_S = abs(xS + 2*xSS + xSA + xSB - S_norm)
err_total = abs(xS + xA + xB + xSS + xAA + xBB + xSA + xSB + xAB - 1)

err_SS = max(0, xSS - xS / 2)
err_AA = max(0, xAA - xA / 2)
err_BB = max(0, xBB - xB / 2)
err_SA = max(0, xSA - min(xS, xA))
err_SB = max(0, xSB - min(xS, xB))
err_AB = max(0, xAB - min(xA, xB))

results.append({
    "xS": xS, "xA": xA, "xB": xB,
    "xSS": xSS, "xAA": xAA, "xBB": xBB,
    "xSA": xSA, "xSB": xSB, "xAB": xAB,
    "err_a": err_a, "err_b": err_b, "err_S": err_S, "err_total": err_total,
    "err_SS": err_SS, "err_AA": err_AA, "err_BB": err_BB,
    "err_SA": err_SA, "err_SB": err_SB, "err_AB": err_AB     })
```

```
# Save results
results_df = pd.DataFrame(results)
output_file = 'results_dry_DES.xlsx'
results_df.to_excel(output_file, index=False)
```

S2.4. Input data

Table S3. The input data (equilibrium constants) for all studied DES systems.

**Code**	**S_name**	**a_name**	**b_name**	**S**	**a**	**b**	**K_SS**	**K_SA**	**K_SB**	**K_AA**	**K_BB**	**K_AB**
ChCl/P2D 1:1	flufenamic acid	choline chloride	1,2-propanediol	0.004	0.498	0.498	2.046E+03	2.396E+06	7.224E+04	2.085E+07	1.229E+08	2.594E+08
ChCl/DEG 1:1	flufenamic acid	choline chloride	diethylene glycol	0.000	0.500	0.500	1.358E+00	1.489E+05	4.727E+01	3.097E+07	7.439E+09	1.306E+10
ChCl/TEG 1:1	flufenamic acid	choline chloride	triethylene glycol	0.005	0.497	0.497	6.499E+02	8.994E+06	3.818E-01	5.388E+07	5.633E-01	4.775E+03
ChCl/B3D 1:1	flufenamic acid	choline chloride	1,3-butanediol	0.005	0.497	0.497	2.236E+03	4.058E+06	7.696E+01	3.174E+07	2.566E+08	1.271E+10
ChCl/GLY 1:1	flufenamic acid	choline chloride	glycerol	0.000	0.500	0.500	2.742E+01	1.030E+05	6.300E+03	1.439E+07	9.943E+07	9.993E+09
ChCl/ETG 1:1	flufenamic acid	choline chloride	ethylene glycol	0.001	0.499	0.499	8.534E+02	5.655E+05	1.216E+04	1.369E+07	6.121E+07	1.291E+09
ChCl/TRG 1:1	flufenamic acid	choline chloride	tetraethylene glycol	0.004	0.498	0.498	1.349E+03	7.453E+06	1.121E+04	4.695E+07	1.283E+05	3.364E+09
Men/P2D 1:1	flufenamic acid	menthol	1,2-propanediol	0.005	0.498	0.498	7.950E+07	2.949E+06	8.062E+06	9.892E+06	3.438E+09	7.771E+06
Men/DEG 1:1	flufenamic acid	menthol	diethylene glycol	0.005	0.497	0.497	4.831E+07	3.640E+06	1.378E+06	6.371E+06	9.776E+11	8.266E+08
Men/TEG 1:1	flufenamic acid	menthol	triethylene glycol	0.009	0.496	0.496	1.305E+08	5.161E+06	3.511E+03	5.478E+06	1.304E+02	1.121E+06
Men/B3D 1:1	flufenamic acid	menthol	1,3-butanediol	0.003	0.498	0.498	3.448E+07	1.702E+06	4.329E+03	9.216E+06	4.990E+10	3.418E+06
Men/GLY 1:1	flufenamic acid	menthol	glycerol	0.005	0.497	0.497	9.090E+07	2.909E+06	7.908E+06	1.225E+07	2.460E+09	2.188E+08
Men/ETG 1:1	flufenamic acid	menthol	ethylene glycol	0.003	0.498	0.498	3.440E+07	2.169E+06	3.866E+06	1.443E+07	1.007E+09	4.362E+06
Men/TRG 1:1	flufenamic acid	menthol	tetraethylene glycol	0.011	0.495	0.495	6.509E+07	3.671E+06	2.947E+06	3.997E+06	9.981E+06	6.318E+07
ChCl/P2D 1:2	flufenamic acid	choline chloride	1,2-propanediol	0.004	0.332	0.664	1.534E+04	1.316E+06	4.805E+05	9.739E+06	6.077E+08	2.461E+08
ChCl/DEG 1:2	flufenamic acid	choline chloride	diethylene glycol	0.000	0.333	0.666	3.342E+01	1.893E+05	9.518E+02	1.784E+07	4.827E+10	1.069E+10
ChCl/TEG 1:2	flufenamic acid	choline chloride	triethylene glycol	0.004	0.332	0.664	7.028E+03	5.943E+06	4.314E+00	3.076E+07	2.711E+00	5.876E+03
ChCl/B3D 1:2	flufenamic acid	choline chloride	1,3-butanediol	0.001	0.333	0.666	2.146E+03	4.237E+05	9.906E+01	1.695E+07	1.845E+09	1.227E+10
ChCl/GLY 1:2	flufenamic acid	choline chloride	glycerol	0.000	0.333	0.667	2.313E+01	4.423E+04	2.960E+04	5.856E+06	4.989E+08	1.198E+10
ChCl/ETG 1:2	flufenamic acid	choline chloride	ethylene glycol	0.001	0.333	0.666	2.387E+02	1.802E+05	4.274E+04	5.787E+06	2.373E+08	1.304E+09
ChCl/TRG 1:2	flufenamic acid	choline chloride	tetraethylene glycol	0.005	0.332	0.663	2.668E+04	5.383E+06	6.152E+04	2.226E+07	7.099E+05	5.366E+09
Men/P2D 1:2	flufenamic acid	menthol	1,2-propanediol	0.002	0.333	0.665	1.564E+07	1.017E+06	4.756E+06	4.871E+06	4.718E+09	6.775E+06

Code	S_name	a_name	b_name	S	a	b	K_SS	K_SA	K_SB	K_AA	K_BB	K_AB
Men/DEG 1:2	flufenamic acid	menthol	diethylene glycol	0.005	0.332	0.663	2.360E+07	2.136E+06	1.118E+06	2.615E+06	1.118E+12	5.406E+08
Men/TEG 1:2	flufenamic acid	menthol	triethylene glycol	0.008	0.331	0.661	6.413E+07	2.832E+06	2.895E+03	2.132E+06	1.499E+02	7.485E+05
Men/B3D 1:2	flufenamic acid	menthol	1,3-butanediol	0.002	0.333	0.665	1.151E+07	7.369E+05	3.036E+03	4.361E+06	6.256E+10	2.545E+06
Men/GLY 1:2	flufenamic acid	menthol	glycerol	0.004	0.332	0.664	3.128E+07	1.339E+06	6.880E+06	6.095E+06	3.391E+09	1.492E+08
Men/ETG 1:2	flufenamic acid	menthol	ethylene glycol	0.002	0.333	0.665	1.469E+07	1.121E+06	3.368E+06	7.535E+06	1.379E+09	4.020E+06
Men/TRG 1:2	flufenamic acid	menthol	tetraethylene glycol	0.011	0.330	0.659	3.541E+07	2.034E+06	2.863E+06	1.478E+06	1.239E+07	4.254E+07
ChCl/P2D 1:3	flufenamic acid	choline chloride	1,2-propanediol	0.004	0.249	0.747	1.437E+05	9.010E+05	1.234E+06	5.345E+06	1.259E+09	1.998E+08
ChCl/DEG 1:3	flufenamic acid	choline chloride	diethylene glycol	0.000	0.250	0.750	2.213E+02	1.369E+05	2.960E+03	1.148E+07	1.149E+11	8.218E+09
ChCl/TEG 1:3	flufenamic acid	choline chloride	triethylene glycol	0.002	0.250	0.749	5.388E+03	2.048E+06	5.826E+00	2.068E+07	5.753E+00	6.395E+03
ChCl/B3D 1:3	flufenamic acid	choline chloride	1,3-butanediol	0.002	0.249	0.748	5.713E+04	5.424E+05	4.653E+02	1.056E+07	4.787E+09	1.003E+10
ChCl/GLY 1:3	flufenamic acid	choline chloride	glycerol	0.000	0.250	0.750	5.374E+01	2.061E+04	4.764E+04	2.949E+06	1.024E+09	1.074E+10
ChCl/ETG 1:3	flufenamic acid	choline chloride	ethylene glycol	0.000	0.250	0.750	3.909E+02	8.007E+04	7.254E+04	2.964E+06	4.428E+08	1.085E+09
ChCl/TRG 1:3	flufenamic acid	choline chloride	tetraethylene glycol	0.002	0.250	0.749	8.969E+03	1.350E+06	4.078E+04	1.364E+07	1.471E+06	6.180E+09
Men/P2D 1:3	flufenamic acid	menthol	1,2-propanediol	0.002	0.250	0.749	8.907E+06	6.259E+05	4.059E+06	2.860E+06	5.234E+09	5.656E+06
Men/DEG 1:3	flufenamic acid	menthol	diethylene glycol	0.004	0.249	0.747	1.533E+07	1.446E+06	9.691E+05	1.390E+06	1.151E+12	3.932E+08
Men/TEG 1:3	flufenamic acid	menthol	triethylene glycol	0.008	0.248	0.744	4.203E+07	1.840E+06	2.499E+03	1.106E+06	1.534E+02	5.485E+05
Men/B3D 1:3	flufenamic acid	menthol	1,3-butanediol	0.002	0.250	0.749	6.583E+06	4.471E+05	2.489E+03	2.513E+06	6.599E+10	1.958E+06
Men/GLY 1:3	flufenamic acid	menthol	glycerol	0.003	0.249	0.748	1.763E+07	8.362E+05	6.237E+06	3.571E+06	3.769E+09	1.103E+08
Men/ETG 1:3	flufenamic acid	menthol	ethylene glycol	0.002	0.249	0.748	1.013E+07	7.720E+05	3.185E+06	4.506E+06	1.522E+09	3.450E+06
Men/TRG 1:3	flufenamic acid	menthol	tetraethylene glycol	0.011	0.247	0.742	2.614E+07	1.373E+06	2.787E+06	7.605E+05	1.327E+07	3.148E+07
Men/P2D 2:1	flufenamic acid	menthol	1,2-propanediol	0.005	0.663	0.332	1.202E+08	4.225E+06	6.725E+06	1.544E+07	1.969E+09	7.023E+06
Men/DEG 2:1	flufenamic acid	menthol	diethylene glycol	0.010	0.660	0.330	2.689E+08	8.886E+06	2.629E+06	1.195E+07	7.107E+11	1.028E+09
Men/TEG 2:1	flufenamic acid	menthol	triethylene glycol	0.010	0.660	0.330	2.878E+08	8.381E+06	4.040E+03	1.071E+07	9.132E+01	1.344E+06
Men/B3D 2:1	flufenamic acid	menthol	1,3-butanediol	0.007	0.662	0.331	1.925E+08	4.820E+06	7.638E+03	1.501E+07	3.094E+10	3.578E+06
Men/GLY 2:1	flufenamic acid	menthol	glycerol	0.012	0.659	0.329	6.597E+08	9.094E+06	1.297E+07	1.862E+07	1.400E+09	2.531E+08

**Code**	**S_name**	**a_name**	**b_name**	**S**	**a**	**b**	**K_SS**	**K_SA**	**K_SB**	**K_AA**	**K_BB**	**K_AB**
Men/ETG 2:1	flufenamic acid	menthol	ethylene glycol	0.006	0.663	0.332	1.435E+08	5.028E+06	5.360E+06	2.072E+07	5.761E+08	3.729E+06
Men/TRG 2:1	flufenamic acid	menthol	tetraethylene glycol	0.011	0.660	0.330	1.395E+08	6.051E+06	2.995E+06	8.520E+06	6.504E+06	7.549E+07
Men/P2D 3:1	flufenamic acid	menthol	1,2-propanediol	0.005	0.746	0.249	1.503E+08	4.970E+06	5.708E+06	1.813E+07	1.259E+09	5.976E+06
Men/DEG 3:1	flufenamic acid	menthol	diethylene glycol	0.007	0.745	0.248	1.765E+08	7.143E+06	1.786E+06	1.516E+07	5.204E+11	1.038E+09
Men/TEG 3:1	flufenamic acid	menthol	triethylene glycol	0.010	0.743	0.248	3.368E+08	9.116E+06	3.580E+03	1.396E+07	6.530E+01	1.330E+06
Men/B3D 3:1	flufenamic acid	menthol	1,3-butanediol	0.006	0.745	0.248	1.762E+08	4.966E+06	5.867E+03	1.790E+07	2.032E+10	3.244E+06
Men/GLY 3:1	flufenamic acid	menthol	glycerol	0.008	0.744	0.248	2.867E+08	6.473E+06	6.235E+06	2.120E+07	8.848E+08	2.458E+08
Men/ETG 3:1	flufenamic acid	menthol	ethylene glycol	0.009	0.744	0.248	3.934E+08	8.576E+06	6.744E+06	2.306E+07	3.679E+08	3.084E+06
Men/TRG 3:1	flufenamic acid	menthol	tetraethylene glycol	0.012	0.741	0.247	2.507E+08	8.161E+06	3.107E+06	1.173E+07	4.460E+06	7.449E+07
